# Cerebral Embolic Protection Devices: Are There Any Indications in Transcatheter Aortic Valve Replacement?

**DOI:** 10.3390/jcm13185471

**Published:** 2024-09-14

**Authors:** Neila Sayah, Ioannis Skalidis, Jules Mesnier, Antoinette Neylon, Mariama Akodad, Anita Asgar

**Affiliations:** 1 Institut Cardiovasculaire Paris-Sud, Hôpital Jacques Cartier, Ramsay-Santé, 91300 Massy, France; a.neylon@icps.com.fr (A.N.); m.akodad@icps.com.fr (M.A.); 2 Department of Cardiology, Lausanne University Hospital (CHUV), 1011 Lausanne, Switzerland; skalidis7@gmail.com; 3 Department of Cardiology, Assistance Publique–Hôpitaux de Paris, Hôpital Bichat, 75018 Paris, France; mesnier.jules@gmail.com; 4 Department of Cardiology, Montreal Heart Institute, Montreal, QC H1T 1C8, Canada; anita.asgar@gmail.com

**Keywords:** aortic stenosis, cerebral embolic protection devices, stroke, transcatheter aortic valve replacement

## Abstract

Stroke following transcatheter aortic valve replacement (TAVR) is a significant and life-threatening adverse event. The vast majority of these incidents occur during the TAVR procedure or within the first 24 h following TAVR, with a notable prevalence of cerebral embolic events. In response to this concern, cerebral embolic protection devices (CEPDs) have been designed to mitigate the risk of peri-procedural ischemic stroke during TAVR. The primary objective of CEPDs is to diminish the intraprocedural burden associated with new silent ischemic brain injuries. Despite the development of several CEPDs, their clinical efficacy remains uncertain. In this review, we delve into a comprehensive analysis of the utilization of CEPDs in patients undergoing TAVR, exploring insights from the existing literature. Additionally, we aim to present future perspectives and discuss the clinical implications associated with the incorporation of CEPDs in TAVR procedures.

## 1. Introduction

Transcatheter aortic valve replacement (TAVR) has transformed the treatment landscape for severe aortic stenosis (AS). Since the first procedure in 2002, TAVR has undergone significant and technical advancements and has demonstrated excellent outcomes. These improvements have facilitated the continuous expansion of TAVR indications to encompass a broad spectrum of surgical risks, extending their application to lower-risk patients with severe AS [1,2]. The accumulated experience has resulted in a decline in complications, such as paravalvular regurgitation and vascular complications. However, one significant concern is the occurrence of stroke, with post-TAVR stroke rates of 1.2–4% [3].

Given its morbidity, avoiding stroke is a critical consideration for patients undergoing TAVR, possibly surpassing even the importance of survival. The incidence of post-TAVR stroke has decreased from the initial pivotal trials to the most recent studies, reflecting improvements in technology, operator experience, and the treatment of lower-risk patients. Despite this decline, stroke rates in real-world registries have plateaued, indicating that stroke remains an unresolved issue, especially given its impact on long-term morbidity and mortality [4,5]. Indeed, post-TAVR stroke is associated with an increased risk of 30-day mortality [6].

Recognizing the importance of stroke prevention, various strategies and devices have been developed to target cerebral embolic protection during TAVR. This review will describe the mechanisms and predictors of post-TAVR stroke as well as the currently available cerebral embolic protection devices (CEPDs) (central illustration). We will discuss their technical aspects, relevant clinical studies, and the overall clinical evidence for their use during TAVR. Additionally, ongoing randomized controlled trials (RCTs) will be described, hopefully offering insights into the evolving landscape of stroke prevention strategies. Ultimately, this review aims to review the literature on the use, benefits, and indications of CEPDs in patients undergoing TAVR, addressing the imperative need to enhance overall TAVR outcomes and mitigate stroke-related morbidity and mortality [7].

## 2. Physiopathology and Etiology

The pathogenesis of stroke following TAVR involves a complex interplay of acute and long-term events. Acute strokes are predominantly associated with procedural elements intrinsic to TAVR. Risk factors for periprocedural stroke include a previous history of stroke, bicuspid aortic valves, arterial/valvular calcium burden, aortic valve pre-/post-dilatation, and valve-in-valve procedures [8] (Table 1). In contrast, longer-term strokes are more intricately linked to the patient’s overall atherosclerotic disease burden and comorbidities, notably atrial fibrillation. They mirror the mechanisms of stroke in the general population, although in a population that is de facto at greater risk. Reduced renal function, diabetes mellitus, history of atrial fibrillation, and increasing age are identified as contributors to the incidence of late (>1 month) strokes [7,8].

The procedural steps of TAVR often lead to the disruption of atheromatous or calcific debris, provoking athero- or thromboembolic events. Approximately 50% of these events occur during the periprocedural phase, with 80% of patients detecting strokes within the first week post-TAVR [8]. Most periprocedural TAVR-related ischemic strokes manifest as a result of embolization of debris forming a nidus for in situ thrombosis once periprocedural anticoagulation wears off. Studies using diffusion-weighted magnetic resonance imaging (DW-MRI) have consistently shown the presence of ‘silent’ ischemic brain lesions in nearly all TAVR patients, highlighting the importance of cerebral embolization mitigation strategies during the procedure [9].

The use of CEPDs has been explored as a means to reduce the risk of embolic stroke during TAVR by capturing these debris. The presence of captured material or debris in the majority of patients undergoing TAVR (90% to 100%), even in low-risk populations, underscores the relevance of understanding the type and size distribution of debris. Larger particles (>1000 μm) have been associated with clinically apparent strokes, and their risk is more pronounced in patients with bicuspid aortic valves. The potential impact of silent cerebral lesions on cognitive function and its long-term prognostic relevance is still a topic of ongoing research, with emerging data indicating that these lesions may not be trivial [6].

The etiology of stroke during and after TAVR involves a multitude of factors, including procedural elements, patient-specific risks, and prosthesis-related considerations. Procedural factors contributing to acute strokes include mechanical disruption of debris during various TAVR steps, suboptimal intraprocedural anticoagulation levels, air embolism, and severe hypotension states. The TAVR procedure, along with transcatheter valve components, induces a prothrombotic environment in the aortic root.

Patient-related risks for stroke encompass increased platelet activation, acute rises in prothrombotic factors, and altered aortic flow dynamics in the neo sinus of the aortic valve. New-onset atrial fibrillation (NOAF) is an independent predictor of delayed strokes, particularly in the first three months after TAVR [10]. Furthermore, delayed strokes may also be linked to factors such as extended time for endothelialization of TAVR valves and the presence of hypo-attenuated leaflet thickening (HALT). The late phase of stroke, occurring beyond 30 days, is often attributed to baseline comorbidities such as hypertension, diabetes, obesity, dyslipidemia, nicotine addiction, and older age.

Operator experience plays a crucial role in reducing the risk of stroke during TAVR procedures. In a study by Salemi A. et al., which examined 8771 transfemoral TAVR procedures carried out by 207 operators between 2012 and 2016, it was observed that patients treated by operators who had performed at least 200 TAVR procedures in the previous year had a significantly lower likelihood of experiencing post-procedural stroke (odds ratio: 0.41; 95% CI: 0.17 to 0.97) and composite adverse events (odds ratio: 0.45; 95% CI: 0.26 to 0.78). These results underscore the need for experienced operators and suggest that higher procedural volumes should be prioritized in TAVR training and hospital practices to optimize patient safety and outcomes [11].

The recognition of silent cerebral embolic lesions and their association with cognitive deterioration poses additional challenges in understanding the etiology of TAVR-related stroke.

## 3. Types of Post-TAVR Strokes

Post-TAVR strokes are classified into three distinct phases, each characterized by its own set of contributing factors.

### 3.1. Early-Phase Strokes (0–72 h)

These strokes are predominantly linked to procedural factors, with micro-debris generated during various TAVR steps being a significant contributor. The acute disruption of atheromatous or calcific debris during valve deployment, balloon aortic valvuloplasty, and post-dilation poses a heightened risk for embolic strokes during this phase. Rapid pacing and diminished blood flow to cerebral watershed areas further compound the risk burden, leading to the majority of strokes manifesting within the initial 72 h post-TAVR. Previous occurrences of stroke, the presence of arterial or valvular calcification, bicuspid aortic valves, as well as aortic valve pre- and post-dilatation procedures, and valve-in-valve interventions have been recognized as risk factors for stroke during procedures. Conversely, diminished renal function, diabetes mellitus, and advancing age have been associated with a higher incidence of delayed stroke [12,13].

### 3.2. Delayed-Phase Strokes (3–30 Days)

Thromboembolism emerges as a notable factor during this phase, with extended time for endothelialization of TAVR valves potentially playing a role. New-onset atrial fibrillation (NOAF) after TAVR becomes an independent predictor of delayed strokes, emphasizing the importance of monitoring and managing atrial fibrillation in the post-TAVR period. In addition, the presence of HALT in TAVR valves has been reported to possibly contribute to a thrombogenic environment, potentially increasing the risk of delayed strokes. However, this relationship is still not clearly established in the literature [14].

### 3.3. Late-Phase Strokes (Beyond 30 Days)

Late-phase strokes are predominantly patient- and disease-related, reflecting the influence of baseline comorbidities. These atherogenic risk factors associated with AS contribute to an increased risk of cerebrovascular events even after the initial phases of TAVR. Understanding and managing these baseline factors are crucial for predicting and preventing late-phase strokes.

## 4. Clinical Trials and Stroke Rates

Stroke rates post-TAVR exhibit notable variability across different clinical trials, influencing the perception of procedural safety and long-term outcomes. Earlier trials, such as the PARTNER I trial, reported higher stroke rates at 30 days for high-risk and inoperable patients undergoing TAVR compared to Surgical Aortic Valve Replacement (SAVR) cohorts (8.3% vs. 4.3%, respectively, *p* = 0.04) [15]. However, recent trials, including PARTNER III, have shown a significant reduction in stroke rates within the TAVR cohort compared to the SAVR cohort (1.2% vs. 3.1%) [3]. Factors contributing to this variance include increased operator experience, advancements in device technology, refined patient selection criteria, and treatment of lower-risk patients.

It is essential to consider the nuances in reported stroke rates. The definition of stroke, the diagnostic evaluation strategy, and the duration of follow-up can significantly impact reported rates. Neurological assessment quality is a critical factor influencing the reported incidence of strokes. Studies emphasizing systematic and optimal neurological evaluation have demonstrated a higher sensitivity in detecting minor strokes or transient ischemic attacks (TIAs), providing a more comprehensive understanding of the impact of TAVR on cerebrovascular events [5].

The impact of antithrombotic regimens on stroke rates among TAVR patients varies depending on their concurrent cardiovascular management needs, such as post-PCI or AF. For patients with PCI history, while dual antiplatelet therapy (DAPT) is generally prescribed to prevent stent thrombosis, the POPular TAVI trial indicated that single antiplatelet therapy (SAPT) led to fewer bleeding events with similar protection against stroke when compared to DAPT [16].

In the context of AF, the choice between non-vitamin K antagonist oral anticoagulants (NOACs) and vitamin K antagonists (VKAs) is influenced by their differential impact on stroke risk. The ENVISAGE-TAVI AF trial suggested that while NOACs were non-inferior to VKAs in preventing strokes, they were associated with higher rates of gastrointestinal bleeding [17]. Conversely, the GALILEO trial raised concerns about increased thromboembolic risks and bleeding with a rivaroxaban regimen versus traditional VKAs. These findings emphasize the need for tailored antithrombotic strategies in TAVR patients, particularly those at high risk for stroke, balancing efficacy in stroke prevention with potential bleeding complications [18].

In conclusion, a detailed examination of post-TAVR stroke types reveals a dynamic interplay between procedural and patient-related factors. Understanding the temporal aspects of stroke occurrence is crucial for targeted preventive strategies. Additionally, interpretation of stroke rates across clinical trials demands a nuanced consideration of study design, patient cohorts, and the quality of neurological assessments employed.

## 5. Types of Embolic Protection Systems

In response to the recognized association between the manipulation of transcatheter valves in the calcified aortic arch and native aortic valve and an elevated risk of emboli with periprocedural acute stroke, CEPDs have emerged as a potential tool to reduce the occurrence of post-TAVR strokes. 

CEPDs address challenges associated with anatomical complexities and atherosclerotic plaques and can be broadly classified into two categories. The first category of devices is designed to capture debris before it reaches the brain arteries, functioning like a net that can either partially or totally entrap embolic material during the procedure. Although the feasibility and safety of these devices have been demonstrated in previous studies, their overall efficacy is still under investigation. The second category is devices employing a deflection system, redirecting debris away from the main arterial branches of the aortic arch toward the descending aorta. Despite not capturing embolic material like filtering systems, deflector devices have shown promising results, with no reported cases of peripheral embolism [19]. 

Devices can be further classified based on the extent of brain protection they provide. Some devices offer partial protection by protecting two of the three primary arteries branching from the aortic arch, including the brachiocephalic trunk, or right common carotid artery, and the left common carotid artery. Other devices provide total protection, encompassing the aforementioned arteries along with the left vertebral artery originating from the left subclavian artery. The left vertebral artery joins the right vertebral artery to create the basilar artery, which plays a crucial role in supplying blood to the posterior part of the circle of Willis. Failing to protect the left subclavian and left vertebral arteries can have important consequences for cerebral protection strategies. Characteristics of CEPDs are provided in Table 2.

### 5.1. Sentinel^®^ Cerebral Protection System

The Sentinel^®^ Cerebral Protection System, developed by Boston Scientific in Marlborough, Massachusetts, stands out as the most extensively studied and widely employed system in TAVR procedures. It was approved by the United States Food and Drug Administration in 2017 and the Conformité Européenne (CE) mark in 2013. The device comprises two interlinked filters housed and delivered with a 6 French (Fr) catheter. Specifically, the proximal filter, ranging from 9 to 15 mm, is deployed in the brachiocephalic trunk, while the distal filter, measuring 6.5 to 10 mm, is positioned in the left common carotid artery. It is important to note that the left vertebral artery, typically originating from the left subclavian artery, remains unprotected.

The percutaneous placement of the Sentinel^®^ device is facilitated through the right radial/brachial artery, utilizing a 0.014-inch coronary guidewire. A comprehensive evaluation of the pre-procedural computed tomography scan is recommended to assess the suitability of the aortic arch and its branches’ anatomy, ruling out excessive tortuosity and calcification.

The Sentinel^®^ device was tested in four RCTs, namely the MRI Investigation in TAVR with Claret (MISTRAL-C) trial, the Claret Embolic Protection and TAVI (CLEAN-TAVI) trial, the US SENTINEL IDE trial, and the Stroke Protection with Sentinel During Transcatheter Aortic Valve Replacement (PROTECTED-TAVR) trial [20,21,22,23]. Notably, the MISTRAL-C and CLEAN-TAVI trials demonstrated fewer new lesions and smaller total lesion volumes in the SENTINEL-protected group, with a higher incidence of neurocognitive deterioration in patients without protection [21,22].

The MISTRAL-C trial, the inaugural RCT evaluating the Sentinel device, involved 65 patients undergoing transfemoral TAVR. The trial aimed to compare the occurrence of new cerebral lesions assessed by DW-MRI and neurocognitive functions before and after TAVR, with and without the Sentinel device. [21] Captured debris were observed in all patients with an embolic protection device (EPD). The EPD group showed a numerical reduction in new cerebral lesions and a lower volume on DW-MRI. Moreover, neurocognitive deterioration is assessed by neurocognitive test evaluation with an assessment of focal neurological deficits through the National Institutes of Health Stroke Scale, degree of autonomy through the modified Rankin Scale, and cognitive status through the Montreal Cognitive Assessment (MoCA) and the Mini Mental State Examination (MMSE). It has been reported that it was less frequent in the EPD group (4% vs. 27%; *p* = 0.017), with a significant decrease in patients with over ten cerebral lesions (20% vs. 0%; *p* = 0.03) [24]. These results highlight the potential benefits of the Sentinel device in reducing cerebral complications during TAVR.

The CLEAN-TAVI trial, which included a randomized cohort of 100 patients, examined new cerebral lesions using DW-MRI two days post-TAVR [22]. In the trial, the EPD group demonstrated a significant reduction in newly occurring brain lesions within both protected territories (4 lesions compared to 10 in the control group; *p* ≤ 0.001) and throughout the entire brain (8 lesions compared to 16 in the control group; *p* = 0.002). Moreover, the EPD group exhibited a smaller median total new lesion volume (466 mm^3^ vs. 800 mm^3^; *p* = 0.02).

The larger-scale US SENTINEL IDE study, involving 363 patients, presented notable findings. While the study did not achieve statistical significance in reducing all-cause stroke at 30 days, the CEPD group exhibited a reduction in stroke within 72 h post-TAVR compared to the unprotected group [23].

The recently published PROTECTED TAVR trial, a randomized, open-label, multicenter, all-comer trial involving 3000 patients, powered for clinical endpoints, revealed that the use of the SENTINEL^®^ device did not manage to substantially reduce the incidence of stroke within 72 h post-TAVR or before hospital discharge compared to the control group (2.3% vs. 2.9%, respectively; *p* = 0.30) [20]. Although the device demonstrated safety and a potential effect on disabling strokes, questions remain on its efficacy and clear clinical benefit, prompting considerations about its practical use and cost-effectiveness, particularly in single-payer health systems.

### 5.2. TriGUARD 3™ Cerebral Protection Device

The TriGUARD™ HDH Embolic Device (TG) from Keystone Heart Ltd. stands as the second most studied device. It offers comprehensive brain protection by covering all three cerebral vessels in the aortic arch. This deflector device comprises a single-use filter made of a fine nickel–titanium alloy mesh, delivered through a contralateral 9 Fr femoral arterial sheath, which can serve as a secondary access for TAVR, eliminating the need for additional vascular access. Under fluoroscopic guidance, the TG device is positioned to cover the ostia of the three main aortic branches and maintained by a stabilizer in the proximal innominate artery.

The safety and efficacy of both first- and second-generation devices have been demonstrated in two RCTs, DEFLECT I and DEFLECT II [25,26]. The pivotal DEFLECT III trial, the first multicenter RCT evaluating the TriGUARD™ in TAVR, included 13 sites across five countries in Europe and Israel [23]. Completed in March 2015, the study randomized patients into an EPD group (*n* = 46) and an unprotected group (*n* = 39). Complete coverage of the cerebral vessel was obtained in 89% of patients. Although the device arm showed numerically lower rates of major adverse cardiovascular and cerebrovascular events (MACCEs) and stroke at 30 days, it did not reach statistical significance (MACCEs at 30 days: 21.7% vs. 30.8%, *p* = 0.34; stroke at 30 days: 2.2% vs. 5.1%, *p* = 0.46). A non-significant reduction in neurological impairment (3.1% vs. 15.4%, *p* = 0.16) and lesion volume was also reported.

The REFLECT I trial, a multicenter RCT with 258 patients, assessed the safety, efficacy, and performance of the TG system in TAVR patients. Though suspended before completing enrollment, the study demonstrated complete coverage in 57.3% of cases and met the primary safety outcome (21.8% vs. 34.4%; *p* < 0.0001). The primary hierarchical efficacy endpoint did not significantly differ between groups [24].

The latest generation, TriGUARD™ 3, was investigated in the REFLECT II trial, which was published in 2021 [25]. In this trial, TAVR patients were randomized to a device arm (*n* = 162) and a control arm (*n* = 121). The primary safety endpoint at 30 days, defined by events such as all-cause mortality, stroke, and major complications, was 15.9% in the cerebral embolic protection device group, significantly better than the historical goal of 34.4% (*p* = 0.0001). The control group had a 7.0% event rate (*p* = 0.11). The primary efficacy endpoint, defined by all-cause mortality, stroke, NIHSS worsening, absence of cerebral ischemic lesions, and lesion volume on DW-MRI at 2–5 days, was −8.58 in the device group vs. 8.08 in the control group (*p* = 0.86).

### 5.3. Other CEPDs

#### 5.3.1. Emblok^®^ Embolic Protection System

The Emblok^®^ Embolic Protection System, developed by Innovative Cardiovascular Solutions in Grand Rapids, MI, USA, is aiming to deliver comprehensive protection to all supraaortic vessels through full circumferential coverage of the aortic arch. This system features an 11 F sheath device that incorporates a 4 Fr pigtail catheter, advanced through femoral access. The filter system, composed of a 125 μm pore-size nitinol, allows simultaneous advancement of the embolic filter and a radiopaque pigtail catheter through femoral access, accommodating various anatomies of the aorta with a diameter of up to 35 mm. In its first-in-human trial involving 20 patients, the Emblok^®^ system demonstrated a remarkable outcome with no MACCE observed at the 30-day follow-up. Although post-procedural DW-MRI indicated the presence of new ischemic brain lesions in 95% of patients, the median new total lesion volume was measured at 199.9 mm^3^ (interquartile range: 83.9–447.5 mm^3^) [26]. This underscores the potential efficacy and safety profile of the Emblok^®^ Embolic Protection System in mitigating adverse events during procedures.

#### 5.3.2. ProtEmbo^®^ Cerebral Protection System

ProtEmbo^®^ (Protembis GmbH, Aachen, Germany) is a deflector device positioned through a 6 Fr left radial access sheath and deployed by unsheathing the self-expanding filter to cover the orifice of all three cerebral vessels, which are commonly heavily calcified in elderly patients. Additionally, it prevents interference with the TAVR delivery system. The device features a low-profile design and a small pore size of 60 μm, effectively protecting the brain from small-sized embolizing particles. The first-generation ProtEmbo^®^ device demonstrated safety and feasibility in the initial PROTEMBO SF trial (*n* = 4 patients; ClinicalTrials.gov: NCT03325283) conducted at two clinical sites in Europe. The PROTEMBO C trial, a prospective, non-randomized, multicenter study (eight sites in Europe), aimed to assess the safety and performance of the second-generation ProtEmbo^®^ Cerebral Protection System. Both primary endpoints were statistically better in the EPD group, with MACCE at 30 days reported at 8.1% (21.3% vs. 25%, *p* = 0.009) and technical success at 94.6% (82.3% vs. 75%, *p* = 0.003) [27,28].

#### 5.3.3. EMBOL-X Device

The EMBOL-X by Edwards Lifesciences in Irvine, California, is a single filter designed for full brain coverage. It is inserted through a mid-sternotomy into the distal part of the ascending aorta. In a single randomized controlled trial with 30 patients undergoing transaortic TAVR, the device showed a non-significant decrease in new cerebral lesions (57% vs. 69%; *p* = 0.70) and significantly smaller lesion volumes in the middle cerebral artery region (33 ± 29 vs. 76 ± 67 mm^3^, *p* = 0.04), as assessed by DW-MRI at 7 days [29]. No neurologic events were observed during the follow-up period. However, its application is limited by the sternotomy required for device implantation.

#### 5.3.4. Embrella Embolic Deflector

The Embrella Embolic Deflector, developed by Edwards Lifesciences, is designed to deflect embolic material during TAVR procedures. It features an oval-shaped nitinol frame with a porous membrane that covers all three major cerebral vessels. The device is delivered through a 6 Fr sheath using the right radial or brachial approach. In the PROTAVI-C trial, which included 52 patients (41 with the device and 11 without), the use of the Embrella device was linked to a significantly greater number of high-intensity transient signals (indicating micro-embolization, assessed by transcranial Doppler) compared to the control group (632 vs. 279, *p* ≤ 0.001) [30]. The study shows that the system did not reduce the number of new lesions but did cut lesion volume by half compared to the control and historical data. This finding aligns with the Triguard device’s results, which also reduced lesion volume but not the number of lesions. All new lesions disappeared within a month after TAVR, and it is considered that smaller lesions are more likely to be transient.

#### 5.3.5. Emboliner^®^ Total Embolic Protection

The Emboliner^®^ Total Embolic Protection, developed by Emboline in Santa Cruz, CA, USA, is currently in investigational use. Engineered to safeguard all three cerebral vessels and the entire body, this device is advanced through a 9 Fr transfemoral sheath, allowing for a 6 Fr pigtail catheter for the TAVR procedure. The device is a cylindrical nitinol mesh filter with a pore size of 125 μm, designed to circumferentially conform to the aortic arch, providing coverage for all three cerebral branch vessels.

Results from the SafePass clinical program, specifically the SafePass 2 trial, were presented at Transcatheter Cardiovascular Therapeutics 2019, demonstrating no adverse events at the 30-day mark with a 100% procedural success rate [31]. While the detailed study results are pending publication, the company has initiated a pivotal trial in the United States in 2023. This trial, aimed at obtaining FDA and CE approval, compares Emboliner^®^ against SENTINEL in 500 TAVR patients, with an additional 40 Emboliner^®^ roll-in cases, utilizing a 1:1 randomization. The primary endpoint is the 30-day combined MACCE rate, encompassing all-cause mortality, stroke, and Stage 3 Acute Kidney Injury (AKI). Results from this pivotal trial are anticipated in December 2024.

## 6. Reasons for Stroke Despite CEPD

Embolic events persist as a challenge even with the implementation of CEPDs during TAVR. Theoretically, CEPDs should prevent embolic materials from reaching the brain, thereby reducing the risk of clinical stroke. However, meta-analyses in the stroke literature indicate no significant difference in the occurrence of new single, multiple, or total lesions. Notably, CEPD usage is associated with a significantly smaller ischemic volume per lesion (standardized mean difference, −0.52; 95% CI, −0.85 to −0.20; *p* = 0.002) and a smaller total lesion volume (standardized mean difference, −0.23; 95% CI, −0.42 to −0.03; *p* = 0.02). The clinical relevance of these radiographic findings remains uncertain, as CEPDs have not been conclusively shown to reduce the incidence of clinically detectable strokes in TAVR procedures [32]. Several factors contribute to this phenomenon. The presence of an unprotected left vertebral artery, originating from the left subclavian artery, and the limitation of having only one available filter size, notably in devices like the Sentinel, may provide insufficient protection against embolic risks in certain anatomical variations. Moreover, the manipulation of structures during both valve procedures and the placement of protection devices introduces an additional layer of complexity. This manipulation may mobilize various structures, serving as potential sources for emboli or debris, which can lead to the event that is intended to be avoided. An additional mechanism for cerebral events involves hemodynamic instability and associated hypotension during TAVR procedures. It is noteworthy that CEPDs may not enhance safety in these situations or even worsen cerebral flow. Factors such as rapid pacing in patients with reduced left ventricular ejection fraction and the use of general anesthesia could contribute to hemodynamic instability, potentially increasing the incidence of neurological events. A recent meta-analysis revealed nuanced differences in stroke distribution but did not find significant variations in stroke severity between patients undergoing TAVR with and without CEPDs. Although CEPDs showed effectiveness in preventing strokes in certain vascular territories like the internal carotid artery and multiple territories, they appeared less effective in reducing vertebrobasilar strokes. Notably, strokes in the middle cerebral artery remained prevalent despite CEPD use, occurring in approximately one-third of cases [33].

The role of CEPDs in current TAVR practices remains contentious, raising ethical and scientific questions. Given the already low stroke rates observed, larger trials are needed to determine if CEPDs can significantly reduce clinical stroke incidents. Moreover, understanding the number needed to treat to prevent one disabling stroke—essential for assessing the cost-effectiveness of CEPD interventions—remains crucial. Additionally, the potential of CEPDs to prevent subclinical strokes and mitigate future cognitive decline remains an unresolved issue awaiting further investigation.

## 7. Clinical Implications

CEPDs have been used during carotid stenting and cardiac surgery for nearly two decades. They have demonstrated their safety and a reduction in neurological events in these settings. In the evolving field of TAVR, there is a growing interest in exploring the potential benefits of CEPDs due to concerns about embolic events during device manipulation in the vasculature, aortic valve, and aortic annulus. The current evidence supporting the use of CEPDs in TAVR is still in the early stages, and, although they are generally considered safe, further investigation is required to confirm their efficacy in preventing significant clinical cerebrovascular events. Future studies with larger populations and robust statistical power are needed, with a particular emphasis on younger populations undergoing TAVR.

One noteworthy challenge for clinicians is the lack of randomized data guiding the appropriate selection of patients for CEPD use in TAVR. Decisions about whether to offer CEPDs routinely, selectively, or not at all remain uncertain, and the outcomes of the PROTECTED-TAVR trial underscore the complexity of this decision-making process.

Patient selection is a critical aspect of CEPD use, and contraindications include significant stenoses, dissections, or aneurysms of the brachiocephalic or carotid arteries. Caution is also advised in cases with significant tortuosity or calcification in the subclavian and arch vessels, as excessive manipulation in challenging anatomy may pose risks of stroke via atheroembolism or vascular dissection.

Despite improvements in transcatheter heart valve design and TAVR techniques, the rate of overt stroke during or soon after TAVR remains consistent at 2–4%. Additionally, emerging evidence suggests the occurrence of cerebral embolization during other left-sided transcatheter heart procedures. There are limited data on the long-term neurocognitive outcomes associated with the use of CEPDs during TAVR. While short-term studies suggest a reduction in subclinical brain infarcts, the potential long-term benefits or risks of CEPDs on cognitive function remain unclear. To fully assess the clinical relevance of CEPDs, extended follow-ups beyond one year are essential. These longer-term studies could provide valuable insights into whether CEPDs help preserve cognitive function or pose unforeseen risks, thus informing their broader use in clinical practice.

## 8. Future Perspectives and Current Practices

To date, the available evidence regarding the routine use of CEPDs in all patients remains insufficient, and there is a notable absence of clear recommendations identifying patients who might derive optimal benefits from such devices. The challenges of drawing definitive conclusions on the clinical benefits of CEPDs are compounded by the low event rates observed in most trials. Numerous ongoing and upcoming studies hold the promise of providing valuable insights for interventional cardiologists in determining the judicious use of CEPDs during TAVR (Table 3).

One of them is the ongoing British Heart Foundation’s PROTECT-TAVI trial (ClinicalTrials.gov ID: NCT02895737), encompassing a substantial cohort of 7730 participants. This open-label, outcome-adjudicated, multicenter, all-comer randomized clinical trial in the UK is designed to randomize patients undergoing TAVR, regardless of the access route, to either a CEPD (utilizing the SENTINEL^®^ device) or no CEPD, without specific exclusion criteria. The primary endpoint is the incidence of stroke at 72 h post-TAVR. With a significant portion of the population already enrolled, an interim analysis is scheduled after reaching 50% of the sample size, with results expected in July 2026 [34].

A pooled patient-level meta-analysis from both the PROTECTED TAVR and BHF PROTECT-TAVI trials (PROSPERO Registry number, CRD42022324160), encompassing over 10,000 TAVR patients, is planned. It will provide more robust insights on the efficacy of the SENTINEL^®^ device in stroke prevention during TAVR. It is important to note that while these trials are essential for the SENTINEL^®^ CEPD, their results do not conclusively extend to CEPDs as a class, necessitating specific clinical validation for all newer generations and devices.

Several other ongoing trials are trying to contribute to the evolving landscape of CEPDs in TAVR. The Emboliner^®^ IDE trial (Protect the Head-to-Head Study (ProtectH2H, ClinicalTrials.gov ID: NCT05684146), expected to conclude by the end of 2024, is a prospective, randomized, open-label, multicenter study comparing the safety and effectiveness of the Emboliner^®^ EPD to the control device (Sentinel^®^ CEPD) in terms of a 30-day composite MACCE rate.

The PROTEMBO trial (NCT05873816), anticipated to conclude in early 2025, is a prospective, multicenter, randomized, controlled study evaluating the safety and efficacy of the ProtEmbo^®^ Cerebral Embolic Protection device compared to a hybrid control (no embolic protection device and the Sentinel^®^ device) in patients with severe symptomatic native AS undergoing TAVR. [35].

The EMBLOK™ Embolic Protection System trial (NCT05295628), set to begin completion in 2025, aims to evaluate the safety, effectiveness, and performance of the EMBLOK™ EPS during TAVR through a randomized comparison with the Sentinel^®^ device.

Lastly, the NAUTILUS study (NCT04704258) focuses on assessing the safety, performance, and treatment effect of the AorticLab FLOWer System in preventing cerebral thromboembolic complications in patients with an indication for TAVR.

The emergence of newer generations of CEPDs, focusing on full-brain and even full-body protection, introduces variables such as access site, sheath size, and mesh pore size. The ultimate protection device should ideally offer comprehensive coverage, ease of delivery and positioning, procedural stability, and, most importantly, clinical effectiveness and safety.

The role of CEPDs in TAVR remains uncertain. While some physicians see them as adding minimal procedure time and risk to patients with potential benefits in reducing stroke risk, others argue that current devices have not consistently lowered clinically significant strokes, adding complexity and cost to TAVR procedures [36].This leaves many clinicians in a middle ground, striving to identify which patients might derive the most benefit from CEPDs [37].

The ongoing challenge lies in identifying specific patient groups that could benefit most from CEPDs, as demonstrated by the PROTECTED-TAVI trial’s subgroup analyses, which did not definitively identify any subgroup that consistently benefited from these devices [38]. Additional obstacles include the added procedural costs, the need for separate access sites potentially increasing vascular complications, and the intricacies of device placement within the aortic arch. Addressing these barriers is crucial for enhancing the routine adoption of newer-generation CEPDs in clinical practice. Nevertheless, the use of the SENTINEL device during TAVR procedures remains uncommon. Data from the STS/ACC TVT registry indicated that it was utilized in only 7.1% of TAVR procedures across 551 sites in the United States between 2018 and 2019. As for reimbursement considerations, while they vary considerably among institutions, the volume of TAVR cases, rather than the reimbursement rate itself, appears to be the primary factor influencing the adoption of CEPDs [39]. However, data from registries must be interpreted with caution and cannot be extrapolated.

## 9. Conclusions

The occurrence of stroke following TAVR represents a devastating and unpredictable adverse event. As indications expand to low-risk patients, mitigating this complication becomes a real challenge. The use of CEPD has proven to be safe with no associated rise in vascular complications. To date, the evidence supporting the systematic use of CEPD remains insufficient.

Further studies are required to better understand the impact of CEPD on clinical outcomes, patient selection, and the potential impact on late neurocognitive impairment after TAVR. A better understanding of the population that could benefit from CEPD as well as the cost effectiveness of stroke prevention devices is also essential to determining the future directions of CEPD development and clinical studies.

## Figures and Tables

**Table 1:** Risk factors of stroke related to TAVR.

Patient-Related Factors	Procedure-Related Factors	Post-Procedural Factors
History of Stroke	Balloon post-dilatation	New-onset atrial fibrillation
Female Sex	Non-transfemoral access	Suboptimal antiplatelet effect
Spontaneous echo contrast	THV embolization/migration	Valve stent incomplete endothelialization
Increased aortic stenosis severity	Prolonged procedure duration	General atherothrombotic burden
Small aortic valve area	Excessive wire and catheter manipulation	
Severely calcified valve	Rapid ventricular pacing	
Impaired left ventricular function	Hemodynamic instability	
High CHA_2_DS_2_-VASc Score		
Bicuspid aortic valve		
Peripheral vascular disease		
Chronic kidney disease		

CHA2DS2-VASc Score: score to predict thromboembolic risk in atrial fibrillation; TAVR: transcatheter aortic valve replacement; THV: transcatheter heart valves.

**Table 2:** Cerebral protection devices: characteristics and main studies.

CEPD	Illustration	Access Site	Mechanism	Major Studies	Study Type	Clinical Findings
**Sentinel^®^ Cerebral Protection System**	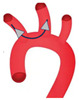	Right radial/brachial artery	Capture	MISTRAL-C	RCT	Stroke disabling 30 d (%): 0 vs. 7
				CLEAN-TAVI	RCT	Stroke non-disabling 7 d (%): 10 vs. 10
				US SENTINEL	RCT	MACCE 30 d (%): 7.3 vs. 9.9 (*p* = 0.4); Stroke 30 d (%): 5.6 vs. 9.1 (*p* = 0.25)
				PROTECTED-TAVR	RCT	Stroke (%): 2.3 vs. 2.9 (*p* = 0.30), disabling stroke: 0.5% vs. 1.3%; (*p* = 0.02), nondisabling stroke: 1.7 vs. 1.5; (*p* = 0.67), mortality (0.5 vs. 0.3)
**TriGUARD 3™ Cerebral Protection Device**	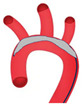	Contralateral femoral artery	Deflector	DEFLECT III	RCT	MACCE composite in hospital (%): 21.7 vs. 30.8 (*p* = 0.34); Stroke composite in hospital (%): 2.2 vs. 5.1 (*p* = 0.46)
				REFLECT I	RCT	MACCE 30 d (%): 21.8 vs. 8.5 RR 2.57 (1.05-6.32; Stroke 30 d (%): 10.7 vs. 6.8 RR 1.58 (0.54–4.59)
				REFLECT II	RCT	MACCE 30 d (%): 15.9 vs. 7 (*p* = 0.115); Stroke 30 d (%): 6.4 vs. 5.3 (*p* = 1.0)
**Emblok^®^ Embolic Protection System**	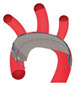	Femoral artery	Capture	Latib et al.	Non-RCT	No MACCE at 30 days
**ProtEmbo^®^ Cerebral Protection System**	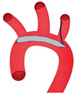	Left radial artery	Deflector	PROTEMBO SF	Non-RCT	Stroke 30 d (%): 0 vs. 0
**EMBOL-X Device**	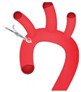	Mid-sternotomy	Capture	TAo-EmbolX	RCT	Stroke 30 d (%): 0 vs. 0
**Embrella Embolic Deflector**	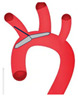	Right radial/brachial artery	Deflector	PROTAVI-C	Non-RCT	Stroke 30 d (%): 4.9 vs. 0 (*p* = 1.0)
**Emboliner^®^ Total Embolic Protection**	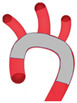	Transfemoral artery	Capture	SafePass 2 trial	Non-RCT	No MACCE at 30 days

CEPD: cerebral embolic protection device; MACCE: major adverse cardiac and cerebrovascular event; RCT: randomized controlled trial.

**Table 3:** Ongoing clinical trials and studies.

	NCT or PROSPERO Registry Number	Design	Country	Device	Primary Endpoint	Expected Completion
**PROTECT-TAVI trial**	NCT02895737	Open-label, outcome-adjucated, multicenter, randomized	UK	Sentinel	Incidence of stroke at 72 h post-TAVR	July 2026
**PROTECTED TAVR and BHF PROTECT-TAVI**	CRD42022324160	Meta-analysis	North America, Europe, Australia	Sentinel	NA	NA
**PotectH2H**	NCT05684146	Prospective, randomized, open-label, multicenter	USA	Emboliner EPD vs. Sentinel	30-day composite MACCE rate	End of 2024
**PROTEMBO trial**	NCT05873816	Prospective, randomized, multicenter, control study	USA	ProtEmbo vs. Sentinel	30-day composite MACCE rate and total new lesion volume	2025
**EMBLOK EPS trial**	NCT05295628	Prospective, randomized, single-blind multicenter, controlled trial	USA	EMBLOK EPS	30-day composite MACCE and debris capture	2025
**NAUTULIUS Study**	NCT04704258	Single arm, prospective, multicenter, non-randomized study	Belgium, Italy	AorticLab FLOWer System	30-day composite MACCE rate and reduction in total volume of new cerebral lesions at 2–5 days	completed

## Abbreviations

AS: aortic stenosis; CEPD: cerebral embolic protection devices; DW-MRI: diffusion-weighted magnetic resonance imaging; MACCE: major adverse cardiovascular and cerebrovascular event; RCT: randomized controlled trials; TAVR: transcatheter aortic valve replacement.
